# The Multifaceted Role of Curcumin in Cancer Prevention and Treatment

**DOI:** 10.3390/molecules20022728

**Published:** 2015-02-05

**Authors:** Muthu K. Shanmugam, Grishma Rane, Madhu Mathi Kanchi, Frank Arfuso, Arunachalam Chinnathambi, M. E. Zayed, Sulaiman Ali Alharbi, Benny K. H. Tan, Alan Prem Kumar, Gautam Sethi

**Affiliations:** 1 Department of Pharmacology, Yong Loo Lin School of Medicine, National University of Singapore, Singapore 117597, Singapore; E-Mails: phcsmk@nus.edu.sg (M.K.S.); a0095676@nus.edu.sg (G.R.); benny_tan@nuhs.edu.sg (B.K.H.T.); 2 Cancer Science Institute of Singapore, Centre for Translational Medicine, 14 Medical Drive, #11-01M, Singapore 117599, Singapore; E-Mail: csikmm@nus.edu.sg; 3 School of Biomedical Sciences, CHIRI Biosciences Research Precinct, Curtin University, Western Australia 6009, Australia; E-Mail: frank.arfuso@uwa.edu.au; 4 Department of Botany and Microbiology, College of Science, King Saud University, Riyadh 11451, Saudi Arabia; E-Mails: dr.arunmicro@gmail.com (A.C.); mozayed@ksu.edu.sa (M.E.Z.); sharbi@ksu.edu.sa (S.A.A.); 5 Department of Biological Sciences, University of North Texas, Denton, TX 76203, USA

**Keywords:** curcumin, inflammation, cancer, NF-κB

## Abstract

Despite significant advances in treatment modalities over the last decade, neither the incidence of the disease nor the mortality due to cancer has altered in the last thirty years. Available anti-cancer drugs exhibit limited efficacy, associated with severe side effects, and are also expensive. Thus identification of pharmacological agents that do not have these disadvantages is required. Curcumin, a polyphenolic compound derived from turmeric (*Curcumin longa*), is one such agent that has been extensively studied over the last three to four decades for its potential anti-inflammatory and/or anti-cancer effects. Curcumin has been found to suppress initiation, progression, and metastasis of a variety of tumors. These anti-cancer effects are predominantly mediated through its negative regulation of various transcription factors, growth factors, inflammatory cytokines, protein kinases, and other oncogenic molecules. It also abrogates proliferation of cancer cells by arresting them at different phases of the cell cycle and/or by inducing their apoptosis. The current review focuses on the diverse molecular targets modulated by curcumin that contribute to its efficacy against various human cancers.

## 1. What Is Curcumin?

**Figure 1:**
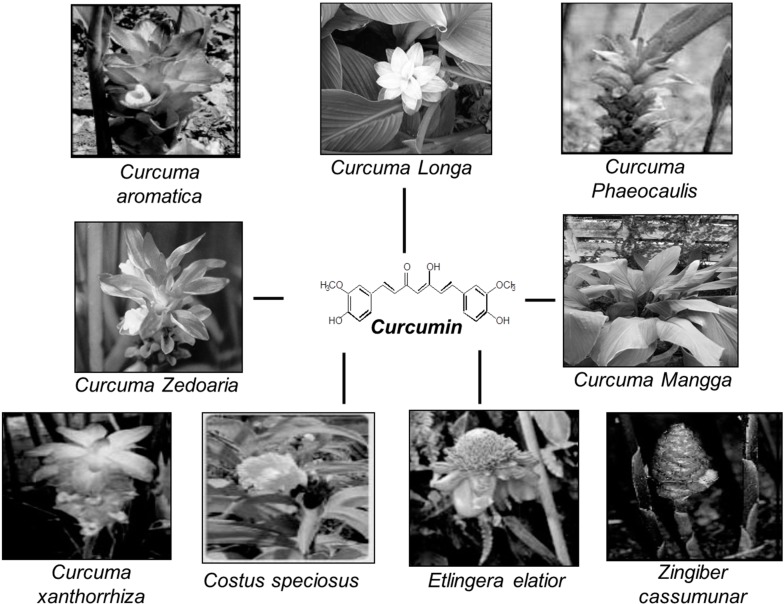
Biological sources and chemical structure of curcumin.

Turmeric is a rhizome from the herb *Curcuma longa Linn*, which was frequently cited in ancient medicinal texts of Ayurveda and in traditional Chinese medicine to be useful for the prevention and cure of a variety of human ailments. Dried turmeric powder is also used in sub-continental cooking and is the main ingredient in all forms of “curry” preparations. Turmeric powder is yellow pigmented and has numerous curcuminoids that include curcumin (77%), demethoxycurcumin (17%), and bisdemethoxycurcumin (3%). Curcumin is a polyphenol (1,7-bis(4-hydroxy-3-methoxyphenyl)-1,6-heptadiene-3,5-dione). Ayurvedic medicine clearly designates curcumin as an effective medicine for various disorders such as asthma, bronchial hyperactivity, allergy, anorexia, coryza, cough, sinusitis, and hepatic disease [1]. Extensive research on curcumin over decades with approximately 6850 publications has provided greater insight into its medicinal and health benefits. There are many reports of its anti-infectious [2], anti-oxidant [3], anti-inflammatory [4,5], hepatoprotective [6], cardioprotective [7], thrombosuppressive [8], anti-arthritic [9], chemopreventive, and anti-carcinogenic [10,11,12] properties. Curcumin has also been shown to modulate multiple cellular molecular targets [13,14]. Several alternative sources of curcumin and its analogues have been reported from other *Curcuma* species such as *Curcuma mangga*, *Curcuma zedoaria*, *Costus speciosus*, *Curcuma xanthorrhiza*, *Curcuma aromatic*, *Curcuma phaeocaulis*, *Etlingera elatior*, and *Zingiber cassumunar* [15]. Figure 1 depicts the various biological sources and chemical structure of curcumin.

## 2. Reported Anti-Cancer Effects of Curcumin

Cancer is a hyper-proliferative disorder where a normal cell loses its cellular homeostasis and begins to constitutively activate a plethora of genes that are involved in cell cycle, invasion, survival, metastasis, and angiogenesis. Curcumin is also a potent anti-inflammatory compound. Based on its distinct chemical properties, curcumin interacts with numerous extracellular and intracellular molecules that are actively involved in cancer initiation and progression, thereby inhibiting cancer progression [16,17,18,19]. Increasing evidence suggests that deregulated inflammatory pathways play a pivotal role in a multitude of chronic diseases, including cancer [20]. The mechanism by which chronic inflammation drives cancer initiation and progression is via increased production of pro-inflammatory mediators, such as cytokines, chemokines, reactive oxygen species (ROS), overexpression of oncogenes, cyclooxygenase (COX-2), matrix metalloproteinase (MMPs), intracellular signaling pathway mediators, transcription factors such as nuclear factor κB (NF-κB), signal transducer and activator of transcription 3 (STAT3), protein kinase B (AKT), and activator protein 1 (AP1) that drive tumor cell proliferation, transformation, invasion, metastasis, angiogenesis, chemoresistance, and radioresistance [17,18,20,21,22,23,24,25,26,27,28].

**Figure 2:**
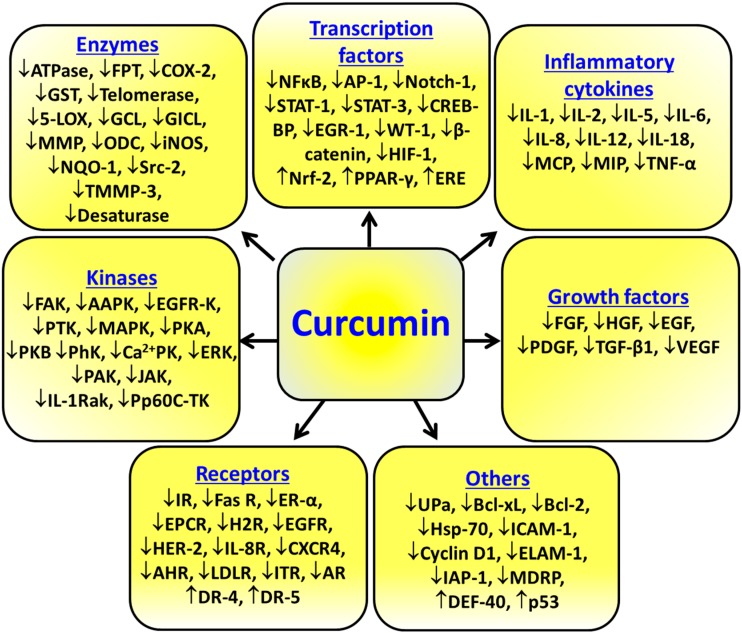
Molecular targets modulated by curcumin. ↓ Down-regulated targets; ↑ Up-regulated targets.

Overall, the various molecular targets modulated by curcumin are summarized in Figure 2. Numerous studies have also reported the inhibitory effects of curcumin on almost all types of tumor cells, such as cancers of the reproductive, digestive, lymphatic and immune, urinary, pulmonary, nervous, skeletal systems, and the skin. The inhibitory concentrations of curcumin have been found to range from 1 μM to 100 μM in these studies [29].

## 3. Molecular Targets Modulated by Curcumin

### 3.1. Transcription Factors

#### 3.1.1. Activator Protein (AP)-1

The transcription factor AP-1 is known to express cancer-relevant genes that activate mitogenic, anti-apoptotic, and pro-angiogenic signals [30,31,32,33]. Members of the MAPK family such as ERK1/2 phosphorylate and activate AP-1 [34], causing up-regulation of CCND1 that encodes cyclin D1 [35,36]. AP-1 is often associated with tumor progression and the high levels of NF-κB and AP-1 expression seen in gliomas is in part responsible for increased chemoresistance and radioresistance [37].

Curcumin has been shown to sensitize human and rat glioma cells to radiation treatment in T98G, U87MG, and T67 cells, and inhibit AP-1 and NF-κB signaling pathways [38]. Curcumin (20 μM) inhibited TPA-stimulated PKC activity in human astroglioma cells and down-regulated pro-angiogenic AP-1 and MMP9 [39]. In human HCT-116 colon cancer cells, curcumin (10–25 μM) inhibited PKC activation by inhibiting the release of Ca^2+^ from the endoplasmic reticulum [40,41]. In another study, curcumin was shown to suppress JNK activation induced by carcinogens [42]. Curcumin abrogated hydrogen peroxide-stimulated proliferation of LnCap prostate cancer cells through the suppression of AP-1 transcription factor [43]. Prusty and Das reported that curcumin down-regulated AP-1 in cervical cancer cells [44]. Therefore, inhibition of PKC activity by curcumin could inhibit neovascularization in tumors by unsettling pro-angiogenic signaling through the ERK-AP-1-MMP-9 pathway [29]. In the DMBA-induced hamster buccal pouch model of carcinogenesis, dietary turmeric (1%) administered for 12 weeks reduced the DMBA-induced tumor burden by down-regulating *Ras* oncogene product p21 [45]. Interestingly, when Hepa1–6 cells were transfected with c-Met-CAT promoter constructs and then stimulated with HGF, there was rapid induction of AP-1 DNA binding activity. When these cells were incubated with curcumin, the c-Met promoter activity was abrogated [46].

#### 3.1.2. Nuclear Factor Kappa B (NF-κB)

Mammalian NF-κB protein comprises five different family members: NF-κB1 (p50/p105), NF-κB 2 (p52/p100), RelA (p65), RelB, and c-Rel. All family members share the common Rel homology domain (RHD: 300aa), which helps in DNA binding and in interaction with IκBs, the intracellular inhibitor of NF-κB [22,47,48,49]. NF-κB can be activated by numerous agents ranging from growth factors, oxidative stress inducers, viruses, gram-negative bacterial products, mitogens, pro-inflammatory cytokines, and environmental stress factors (such as ultraviolet light, H_2_O_2_, cigarette smoke, and asbestos), chemotherapeutic agents, and gamma radiation [50,51,52]. Upon activation by pro-inflammatory cytokines, such as tumor-necrosis factor-alpha (TNF-α) and interleukin 1β (IL-1β), IκBs can be phosphorylated at two equivalent serine residues, S19 and S23, by both IKKα and β [18,47,53]. The phosphorylated NF-κB then migrates to the nucleus and initiates gene transcription of various oncogenic genes that suppress apoptosis but induce cell proliferation and transformation, invasion, metastasis, chemoresistance, radioresistance, and angiogenesis [22,24,50,54,55]. Interestingly, endogenous WIP1 phosphatase was show to be a negative regulator of NF-κB [56]. The active constitutive form of NF-κB has been reported to occur in almost all cancers, and the ability of curcumin to suppress activation of NF-κB is of particular interest in cancer therapy [13,57].

The anti-cancer activity of curcumin was first demonstrated in the 1980’s by Kuttan and colleagues [58,59]. In 1995, Singh and Aggarwal showed that curcumin exhibits its anti-inflammatory activity by suppressing NF-κB activity [60]. Research on curcumin has significantly increased over the years and approximately 7000 articles have been published till date, as indicated by NIH PubMed database [61]. Curcumin efficiently suppressed activation of NF-κB induced by various stimuli such as phorbol ester, TNF-α, and hydrogen peroxide [60]. Subsequently, other researchers showed that curcumin inhibited the NF-κB pathway upstream kinases, IKKα and IKKβ [62,63]. In another study, they showed that curcumin inhibited cigarette smoke-induced NF-κB activation in human lung epithelial cells [64], and also inhibited constitutive activation in head and neck cancer, multiple myeloma, and mantle cell lymphoma [65]. Interestingly, curcumin abrogated paclitaxel-induced NF-κB activation in breast cancer cells and inhibited lung metastasis in a breast cancer nude mouse model [66]. Recent studies have shown that down-regulation of NF-κB led to suppressed expression of cyclin D1, COX-2, MMP9, and pro-MMP2 [67,68].

Curcumin inhibits the Notch signaling pathway in pancreatic cancer cells [69], and has been shown to be a potent proteasomal inhibitor [70], inhibiting the 20S proteasome and inducing degradation of IκBα in colon cancers [71]. In addition, curcumin inhibited NF-κB -induced production of CXCL1 and CXCL2 in breast cancer cells [72]. Curcumin can abrogate NF-κB pathway in multiple cancer cells [73], including breast cancer [74,75,76,77,78,79,80,81], colorectal cancer [82,83], human oral squamous carcinoma [84], human bladder cancer [85,86], cutaneous T-cell lymphoma [87,88], pancreatic cancer [89], head and neck squamous cell carcinoma [90,91], adenoid cystic carcinoma [92], glioblastoma [93,94], human tongue squamous cell carcinoma [95], human biliary cancer [96], medulloblastoma [97], gastric cancer [98], lymphoma [99,100], Myeloid-derived suppressor cells [101], ovarian cancer [102], T-cell and NFAT activation [103], rhabdomyosarcoma [104,105], esophageal adenocarcinoma [106,107], esophageal squamous cell carcinoma [108,109], human epidermoid carcinoma [110], prostate cancer [111,112,113], non-Hodgkin’s lymphoma [114], Hodgkin’s lymphoma [115], human Burkitt’s lymphoma [116], and thyroid carcinoma [117], exemplifying curcumin as a potential chemopreventive, oncostatic, and anti-metastatic agent.

#### 3.1.3. Peroxisome Proliferator-Associated Receptor Gamma

Peroxisome proliferator-associated receptor gamma (PPAR-γ) is a member of the nuclear receptor family [118]. Activation of PPAR-γ has been identified to be involved in inducing differentiation and inhibiting proliferation of cancer cells [119,120,121,122]. Curcumin was reported to activate PPAR-γ and inhibited the growth of Moser cells, with subsequent inhibition of cyclin D1 and EGFR gene expression [123]. In another study, curcumin activated PPAR-γ in cholangiocarcinoma cell lines (KKU100, KKU-M156, and KKU-M213) and induced apoptosis in these cell lines [124]. Curcumin significantly inhibited proliferation in Eker rat-derived uterine leiomyoma cell lines (ELT-3) via activation of PPARγ, where curcumin acted as a PPARγ ligand and, in the presence of a PPARγ antagonist, the inhibitory effect of curcumin was reversed [125].

#### 3.1.4. Signal Transducer and Activator of Transcription (STAT)

The STAT family consists of seven members: STAT1, STAT2, STAT3, STAT4, STAT5a, STAT5b, and STAT6. They range in size from 750 to 850 amino acids [28,126,127]. Among other STAT family proteins, STAT3 has received considerable attention during the last decade since it is a convergent point for a number of oncogenic signaling pathways, and controls intra-cellular signal transduction pathways of several pro-inflammatory cytokines and growth factors [128,129]. STAT3 can be activated by IL-6, EGFR, PDGF, leukemia inhibitory factor (LIF), oncostatin M, and the ciliary neurotrophic factor (CNTF) family of cytokines, which are all known to mediate their signal through the gp130 protein [130,131,132,133]. IL-6 is a key event in tumorigenesis, with high levels associated with hepatocellular carcinoma (HCC) [134].

Constitutively activated STAT3 has been implicated in multiple cancers such as head and neck cancer [135], leukemias [136], lymphomas [137], and multiple myeloma [138], making it a potential target for cancer therapy. Under normal physiological conditions, STAT3 contributes to cell survival and growth, and prevents apoptosis by up-regulating the expression of anti-apoptotic proteins such as Bcl-2 and Bcl-xL. In addition, STAT3 also activates VEGF expression thereby increasing angiogenesis. In a study by Bharti *et al.*, curcumin was shown to inhibit interleukin IL-6 induced STAT3 phosphorylation and consequent STAT3 nuclear translocation in multiple myeloma cells [138]. Curcumin was more efficient and more potent than the well-characterized JAK2 inhibitor AG490. In addition, dexamethasone-resistant multiple myeloma cells were found to be sensitive to curcumin [138]. In a subsequent follow-up study, Bharti *et al.*, reconfirmed their earlier findings by testing the effect of curcumin on CD138^+^ cells derived from multiple myeloma patients. Curcumin substantially inhibited constitutively active STAT3 in these cells [138,139]. In primary effusion lymphoma cells, curcumin inhibited cell proliferation and induced caspase-dependent apoptosis in a dose-dependent manner via suppression of the JAK/STAT3 pathway [140].

In the HTLV-I-transformed T cell leukemia lines, MT-2, HuT-102, and SLB-1, which express constitutively phosphorylated JAK2, TYK2, STAT3, and STAT5 signaling proteins, curcumin induced a dose-dependent decrease in JAK and STAT phosphorylation, resulting in the induction of growth-arrest and apoptosis in T cell leukemia [141]. In another study by Charkravarti *et al.*, curcumin inhibited constitutive and IL-6 induced STAT3 phosphorylation in a dose and time dependent manner and induced apoptosis of HNSCC cells [142]. Attenuation of JAK-STAT3 phosphorylation by curcumin in K562 chronic leukemia cells resulted in suppression of *JAK2*, *cyclin D1*, and *v-src* gene expression [143]. In multidrug-resistant breast cancer model, MCF-7 and MCF-7R (which lacks estrogen receptor alpha [ERα] and overexpresses p-glycoprotein, different IAPs ([inhibitor of apoptosis proteins] and COX-2, curcumin inhibited cell proliferation and induced apoptosis, and the effect was more potent in the multidrug-resistant MCF-7R compared to its parental MCF7 cells [144]. In a Hodgkin’s lymphoma cell line, curcumin induced cell-cycle arrest and induced apoptosis [145]. In numerous studies using various cancer cell lines, curcumin was shown to inhibit cellular proliferation by down-regulating the JAK-STAT3 pathway in chronic lymphocytic leukemia B cells [146], human multiple myeloma U266 cells [147], pancreatic cancer cells [148,149], ovarian and endometrial cancer cells [150,151], melanoma cells [152,153], cutaneous T-lymphoma cells [87], malignant gliomas [154,155], myeloid-derived suppressor cells [101], lung cancer [156,157], and hepatocellular carcinoma [158]. Thus, curcumin presents proven potential as an effective inhibitor of STAT3 phosphorylation and of downstream gene transcription.

#### 3.1.5. Wnt/β-catenin

Signaling by Wnt glycolipoproteins is one of the fundamental mechanisms that direct cell proliferation and tissue homeostasis [159]. Mutations in the Wnt pathway are often linked to cancer and other diseases [160]. Wnt, in association with the transcriptional co-activator β-catenin, controls the development of gene expression programs. De-regulated Wnt/β-catenin is commonly observed in colon cancer [161]. Curcumin treatment inhibits both Wnt and cell-cell adhesion pathways, resulting in induction of apoptosis in HCT-116 colon cancer cells [162,163,164]. In a study using colon cancer cells, it was shown that curcumin suppresses the Wnt/β-catenin pathway through down-regulation of the transcriptional co-activator p300 [165]. In human breast cancer cells, curcumin was found to effectively inhibit the expression of several Wnt/β-catenin pathway components such as disheveled, beta-catenin, cyclin D1, and slug in both MCF-7 and MDA-MB-231 breast cancer cell lines [166]. Aberrant Wnt/β-catenin signaling promotes osteosarcoma tumorigenesis and metastasis. Treatment with curcumin reduced osteosarcoma cell proliferation, invasion and migration, and induced apoptosis by suppressing the Wnt/β-catenin pathway [167]. In LnCap prostate cancer cells, curcumin treatment inhibited androgen receptor expression and inhibited Wnt/β-catenin both in cytoplasmic as well as nuclear extracts and in whole cell lysates [168]. In yet another study using androgen-independent prostate cancer cells, curcumin was found to decrease the level of Tcf-4, CBP, and p300 proteins that are implicated in the Wnt transcriptional complex, leading to the decrease of ß-catenin/Tcf-4 transcriptional activity and of the expression of β-catenin target genes (*cyclin D1* and *c-myc*) [169]. In another study, curcumin was found to negatively regulate Wnt/β-catenin pathway by activating protein kinase D1 in prostate cancer cells [170]. In human hepatocellular carcinoma, curcumin inhibits proliferation and induces apoptosis by suppressing the Wnt/β-catenin pathway [171]. In a recent study using meduloblastoma cell lines, attenuation of the Wnt/β‑catenin pathway by curcumin was due to the suppression of nuclear β-catenin [172], and in non-small cell lung cancer, curcumin inhibited metastasis-associated protein 1-mediated inactivation of the Wnt/β-catenin pathway [173].

#### 3.1.6. Nrf-2

Upon redox-dependent stimuli, the transcription factor Nrf-2-Keap interaction enables Nrf-2 to translocate to the nucleus and bind to the antioxidant-response element (ARE) and initiate transcription of genes encoding for antioxidant and detoxifying enzymes via heme oxygenase-1 (HO-1), NADPH quinone oxidoreductase-1, and glutathione [174,175]. Several strategies for induction of Nrf-2 activity to prevent cancer development have been employed over the last couple of years [176]. Curcumin treatment was shown to increase the expression of transcription factor Nrf-2, a key transcriptional regulator of antioxidant and detoxifying enzymes in renal epithelial cells [177]. Nrf-2 has been shown to be necessary for the up-regulation of genes involved in oxidative stress, such as glutathione *S*-transferase superoxide dismutase-containing ARE [178]. Curcumin activates ARE-mediated gene expression in human monocytes via PKC delta, upstream of p38 and Nrf-2 [179].

In human hepatocytes, curcumin induced ROS generation, activated Nrf-2 and MAP kinases, inhibited phosphatase activity in hepatocytes and induced HO-1 [180]. Curcumin showed chemopreventive effect against prostate cancer in TRAMP C1 cells through epigenetic modification of the Nrf-2 gene, with subsequent induction of the Nrf-2-mediated anti-oxidative stress cellular defense pathway [181]. Over-expression of Flap endonuclease 1 (Fen1), a DNA repair-specific nuclease, is involved in the development of breast cancer. In another recent study, Chen *et al.*, recently showed that curcumin can inhibit breast cancer cell proliferation through Nrf-2 mediated down-regulation of FEN-1 expression [182].

#### 3.1.7. Pro-Inflammatory Cytokines

##### Tumor Necrosis Factor-alpha (TNF-α) and Interleukins

TNF-α has been shown to mediate tumor initiation, promotion, and metastasis [183,184]. The pro-inflammatory effects of TNF-α are due primarily to its ability to activate NF-κB. Almost all cell types, when exposed to TNF-α, activate NF-κB, leading to expression of inflammatory genes such as COX-2, 5-LOX, cell adhesion molecules, inflammatory cytokines, chemokines, and inducible nitric oxide synthase [185]. TNF-α also functions as a growth factor for most tumor cells [186,187]. Curcumin suppresses the expression of TNF-α at both the transcriptional and post-transcriptional levels. TNF-α increased the expression of intracellular adhesion molecule-1 (ICAM-1), vascular cell adhesion molecule-1 (VCAM-1), and endothelial leukocyte adhesion molecule-1 (ELAM-1) in human umbilical vein endothelial cells. Pre-treatment with curcumin inhibited the TNF-α induced adhesion of monocytes to vascular endothelial cells [188]. Curcumin (10 and 100 micromol/l) inhibited TNF-α secretion from trypsin or activating peptide-stimulated human leukemic mast cells [189]. The combination of curcumin and sulforaphane inhibited the inflammatory markers iNOS, COX-2, prostaglandin E2 (PGE2), TNF-α, and interleukin-1 (IL-1) [190]. In another study, curcumin was shown to down-regulate the expression of TNF-α mRNA in K562 leukemia cells [191].

In an orthotopic mouse model of lung cancer using intravenously injected Lewis lung carcinoma (LLC) cells, 5% w/w dietary curcumin exerted physiological changes in lung tissues by significantly decreasing LPS-induced TNF-α production in lungs [192]. Orally bioavailable curcumin blocks TNF-α action and secretion in *in vitro* models, in animal models, and in humans [193]. In breast epithelial and breast cancer cells, curcumin reversed TNF-α induced Warburg-like metabolism [194]. The anti-cancer effect of curcumin against lymphoma has been reported previously. However, in this study they reported that long-term use of curcumin may attenuate cancer progression via the down-regulation of TNF-α and of IL-6 modulated by E26 transformation-specific protein (ETS) and NF-κB [195]. In general, various plant-derived polyphenols were shown to suppress TNF-α activated inflammatory pathways in both *in vitro* and *in vivo* models of cancer [196]. Curcumin can inhibit the expression of both TNF-α mRNA and TNF-α protein in mantle cell lymphoma cell lines. Suppression of TNF-α by curcumin led to the abrogation of NF-κB activation and cell proliferation, as was the case when TNF-α secretion was neutralized using an anti- TNF-α antibody [65]. Curcumin prevented both cadmium-induced IL-6 and IL-8 secretion by human airway epithelial cells [197].

#### 3.1.8. Inflammatory Enzymes

##### Inducible Nitric Oxide Synthase (iNOS), Cyclooxygenase-2 (COX-2), Lipooxygenase (LOX)

COX-2, LOX, and iNOS are important enzymes that mediate inflammatory processes. Curcumin is a known, potent anti-inflammatory agent that prevents tumor progression and exerts chemopreventive effects on carcinogenesis [198,199]. Inducible nitric oxide synthase (iNOS) is an inflammation-induced enzyme that catalyzes the production of nitric oxide (NO), a molecule that may lead to carcinogenesis. Curcumin inhibited iNOS expression in *ex vivo* cultured BALB/c mouse peritoneal macrophages in a concentration-dependent manner [200]. Curcumin suppressed lipopolysaccharide (LPS)-induced expression of iNOS and COX-2 and prevented colon carcinogenesis [201]. Curcumin reduced cholangiocarcinogenesis in hamsters via reduction in the expression of pro-inflammatory proteins such as COX-2 and iNOS [124]. In combination with polyunsaturated fatty acids, curcumin produced a synergistic effect and inhibited the proliferation of RAW 264.7 macrophages by suppressing iNOS, COX-2, 5-lipoxygenase (5-LOX), and cPLA(2) [202].

#### 3.1.9. Oncogenic Kinases

Functional activation of key protein kinases, including IκB kinases and mitogen-activated protein kinases (MAPKs) such as p38 MAPK, JNK1/2, and extracellular signal-regulated kinase 1/2 (ERK1/2), all of which are involved in activating key transcription factors such as NF-κB and AP-1, serve as important target molecules for cancer prevention and therapy [203]. Protein kinases and growth factors are also targets of curcumin, which has been shown to down-regulate the activity of ERK1/2 in pancreatic and lung adenocarcinoma cells [204]. Curcumin has also been shown to inhibit the PI3 kinase/AKT pathway in malignant glioma cells [205]. Curcumin inhibits a plethora of kinases including phosphorylase kinase, protein kinase C, protamine kinase, auto-phosphorylation-activated protein kinase, and pp60c-src tyrosine kinase [13,18]. Curcumin-induced apoptosis of neutrophils was mediated via activation of p38 MAPK and induction of caspase-3 [206]. In experimental colitis, curcumin inhibited p38 MAPK [207]. In another study by Chen *et al.*, curcumin suppressed JNK activation induced by various stimulants such as ionomycin, anisomycin, phorbolmyristate acetate, γ-radiation, TNF-α, UV-C, and sodium orthovanadate [42].

### 3.2. Growth Factor Induced Signaling Cascades

ErbB-2 (avian erythroblastosis oncogene B) or HER2/neu is a member of the epidermal growth factor receptor (EGFR) family and plays an important role in the pathogenesis of breast cancer. It is a cell membrane tyrosine kinase receptor that binds to growth factors and mediates cell proliferation and differentiation. HER2/neu is over-expressed in breast cancer [208], and both HER2 and EGF receptors stimulate proliferation of breast cancer cells. Curcumin suppressed EGFR signaling in prostate cancer cells by inhibiting ligand-induced activation of EGFR and its intrinsic tyrosine kinase activity [209]. In colorectal cancer cells, Caco-2 and HT-29, curcumin down-regulated the transcription factor, Egr1, and the expression of EGFR, thereby inhibiting colorectal cancer cell growth [10]. In non-small cell lung cancer (NSCLC), activating mutations of EGFR are responsive to erlotinib, an EGFR antagonist, while somatic mutations of EGFR are non-responsive. Curcumin significantly increased the cytotoxicity of erlotinib -resistant H1975 and H1650 NSCLC cells, and enhanced erlotinib-induced apoptosis, down-regulated the expression of EGFR, p-EGFR, and survivin in erlotinib-resistant NSCLC cells [210].

Curcumin improved the efficiency of gefitinib in the drug-resistant NSCLC cells both *in vitro* and *in vivo* by inducing EGFR degradation and modulating p38 activation [211]. In another study using human bronchial epithelial (HBE) Beas-2B cells and lung cancer A549 cells, curcumin decreased EGFR expression [212]. Curcumin modulated the migratory and invasive ability of the highly metastatic mouse hepatoma Hca-F cells, through a novel mechanism involving inactivation of Cav-1 and EGFR signaling pathways [213]. Curcumin was shown to inhibit functional interaction between integrin α6β4 and growth factor receptor, EGFR, a key event in breast carcinoma cell motility and invasion [214]. In colon cancer cells treated with 5-FU plus oxaliplatin (FOLFOX), the addition of curcumin down-regulated activation of EGFR, HER-2, IGF-1R, and AKT [215,216]. Curcumin attenuated EGF-induced aquaporin water channels in cells from the human ovarian cancer cell line, CaOV3, and thereby inhibiting cell migration and metastatic potential [217].

### 3.3. Other Protein Kinases and Inflammatory Mediators

#### 3.3.1. Cyclin D1

Cyclin D1 is a subunit of Cdk4 and Cdk6, and is over-expressed in a multitude of cancers such as lung, liver, head and neck, prostate, breast, colon, and multiple myeloma cells [13]. The expression of cyclin D1 is mainly controlled through the NF-κB pathway. Suppression of the NF-κB pathway leads to suppression of cell cycle progression mediated by cyclin D1. Curcumin was shown to down-regulate cyclin D1 [218]. Curcumin inhibited cell proliferation and induced G2/M cell cycle arrest in HCT-116 cells. Immunoblotting analysis showed that cyclin D and E levels declined while cyclin B levels were unchanged. Curcumin inhibited cell proliferation and induced G2/M arrest in HCT-116 cells. Investigation of the levels of cyclins E, D, and B by immunoblotting analysis showed that cyclin B level was unaffected, whereas cyclin D and E levels declined with curcumin treatment [219]. In immortalized human endothelial-like cells (ECV304), curcumin inhibited cell proliferation and induced G0/G1 and/or G2/M phase cell cycle arrest, up-regulated cyclin-dependent kinases, p21WAF1/CIP1, p27KIP1, and p53, and slightly down-regulated cyclin B1 and cdc2 [220].

Curcumin can also down-regulate p21WAF1/CIP1 expression in prostate cancer cells [221]. In four MYCN-amplified neuroblastoma cell lines, with wild-type or mutant p53, curcumin induced p21WAF1/CIP1 [222]. In another study, curcumin was reported to up-regulate cyclin dependent kinase inhibitors p21 and p27 in multiple tumor cell lines [16]. Moreover, Schaaf *et al*., (2009) found that curcumin suppressed the proliferation of 25 human pituitary adenocarcinoma cells by inhibiting cyclin D1 and cyclin-dependent kinase 4 and induced G2/M cell cycle arrest [223]. Curcumin enhanced the anti-proliferative effect of mitomycin C and induced cell cycle arrest, inhibited cyclin D1, cyclin E, cyclin A, cyclin-dependent kinase 2 (CDK2) and CDK4, along with the induction of the cell cycle inhibitors p21 and p27 in MCF-7 cells [224]. In lung cancer cells, curcumin treatment inhibited cell proliferation, which was associated with up-regulation of p27 and p21, and down-regulation of cyclin D1 [225].

#### 3.3.2. p53

p53 inactivation and NF-κB activation are commonly observed in a variety of cancers and play an important role in cancer progression [226,227]. It has been shown that curcumin possesses dual activities such as activation of p53 and inhibition of NF-κB thereby inducing apoptosis. Curcumin induces p53-dependent apoptosis in basal cell carcinoma [228]. Zheng *et al.*, showed that, curcumin up-regulated p53 in human melanoma cells [68]. In the breast cancer cell line MCF7, curcumin-induced apoptosis was accompanied by an increase in p53 level, increased DNA-binding activity, and delayed increase in the effector Bax expression [229]. In a neuroblastoma cell line, curcumin treatment transiently up-regulated p53 expression and induced nuclear translocation of p53, followed by induction of p21 (WAF-1/CIP-1) and Bax expression [222]. In mammary epithelial carcinoma cells, curcumin was reported to induce apoptosis by up-regulating p53 [230]. Curcumin up-regulated p53 in HT-29 colon cancer cells [231], and, in combination with oxaliplatin, produced a 16-fold induction of p53 protein in both p53wt and p53 mutant colorectal tumors [232]. Curcumin also enhanced expression of p53 molecule in tumor tissue, and modulated the apoptotic pathway in colorectal cancer cells [233], ovarian cancer [234], cisplatin-resistant ovarian cancer [235] and induced the expression of FOXO3a and p53 in nasopharyngeal carcinoma [236] by serine phosphorylation of p53 in a concentration- and time-dependent manner. Interestingly, curcumin down-regulated MDM2 independently of p53 in PC3 prostate cancer cells [237], while it was shown to up-regulate p53 protein expression in LnCap prostate cancer cells [238]. Curcumin induced apoptosis of human glioma cells in a p53-dependent manner followed by induction of p21 WAF-1/CIP-1 and ING4 [239]. In another study using human leukemia cells, curcumin-induced apoptosis was mediated by up-regulation of p53 and prolonged the life span of tumor bearing mice [240]. In bile duct cancer, curcumin increased p53 and Bax protein levels and was associated with marked oxidative stress and apoptosis [241]. In human renal carcinoma Caki cells, curcumin enhanced dual PI3K/Akt and mTOR inhibitor NVP-BEZ235-induced apoptosis through p53-dependent Bcl-2 mRNA down-regulation at the transcriptional level and Mcl-1 protein down-regulation at the post-transcriptional level [242]. Similarly, a small molecule compound nutlin-3 showed dual activity by simultaneously activating p53 and suppressing NF-κB [243]. Interestingly, curcumin was found to down-regulate p53 expression in lung cancer cells and induced apoptosis independent of p53 [244]. Bush JA *et al.*, (2001) reported a different mechanism of action of curcumin. In their study, they showed that curcumin induced apoptosis in human melanoma cells through the Fas/caspase-8 pathway independent of p53, and also that curcumin did not induce p53 in these cells [245]. In both MDA-MB-231 breast cancer cells [246], and in HCT-15 colorectal cancer cell line, curcumin was found to down-regulate p53 expression [247]. Curcumin induced p53-independent apoptosis in ovarian carcinoma cells that involved p38 MAPK activation [248]. Another study demonstrated that when curcumin was used in combination with trichostatin, it down-regulated p53 expression in breast cancer cells [249]. These contrasting effects of activation or suppression of p53 in various cancer cells by curcumin warrant further investigation.

#### 3.3.3. Adhesion Molecules

Leukocyte recruitment by endothelial cells and their following movement from the vasculature into the tissue play major roles in inflammation-driven cancers. Treatment of vascular endothelial cells with TNF-α induced adhesion of monocytes to the vasculature due to increased expression of intracellular adhesion molecule-1 (ICAM-1), vascular cell adhesion molecule-1 (VCAM-1), and endothelial leukocyte adhesion molecule-1 (ELAM-1). Pre-treatment of vascular cells with curcumin inhibited monocyte adhesion with a concomitant decrease in ICAM-1, VCAM-1, and ELAM-1 expression, and inhibited cellular migration and invasion of SK-Hep-1 hepatocarcinoma cells [188,250] and small cell lung cancer cells [157]. Matrix metalloproteinases (MMPs) are metal-dependent endopeptidases capable of degrading components of the extracellular matrix. MMPs are involved in chronic diseases such as cancers [251]. In human non-small cell lung carcinoma cells, curcumin suppressed cigarette smoke-induced MMP9 secretion by inhibiting the NF-κB pathway [64]. Curcumin also inhibited MMP9 in a variety of cancers such as prostate cancer [113], colon cancer [252], human promyelocytic leukemia [253], breast cancer [254], human laryngeal squamous carcinoma [255], metastatic colorectal cancer [256], U373 cells [257], lung cancer [258,259], nasopharyngeal carcinoma [260] and liver metastasis from colorectal cancer [261].

### 3.4. In Vivo Studies

**Figure 3:**
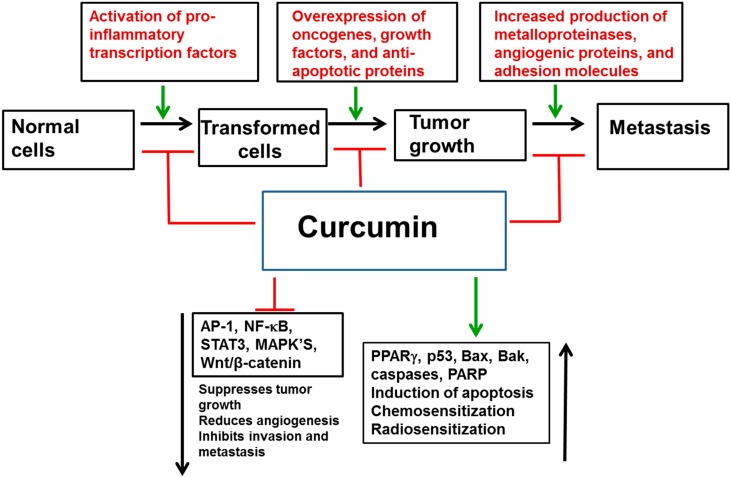
Potential anti-cancer functions of curcumin.

Curcumin has been tested in numerous animal models of cancer and on almost all types of organ-specific cancers, including breast [262,263], oral [264], head and neck [265,266], hepatocellular carcinoma [267], pancreatic [268,269], prostate [270,271,272], colon [273,274], gastric [275], as well as multi-drug resistant cancer cells [276], cancer stem cells [277], and in a variety of other cancers as well as in chemoprevention, and chemoresistance [17,278,279,280,281,282,283]. The *in vivo* molecular targets of curcumin have been excellently reviewed previously [284,285,286,287,288]. Figure 3 clearly illustrates the potential role of curcumin in negatively regulation tumor initiation, progression and metastasis.

### 3.5. Clinical Trials with Curcumin

Curcumin as a therapeutic has been used for thousands of years but it was not until 1937 that Oppenheimer carried out the first clinical trial of curcumin against cholecystitis [61]. However, it was not until 1987 that the anti-cancer property of curcumin was evidenced by Kuttan *et al*. [59], where topical application of curcumin proved to be effective against external cancerous lesions. The multifaceted role of curcumin has been demonstrated in various human clinical trials over the past quarter century against many diseases including cancer, cardiovascular disease, diabetes, oral lichen planus, arthritis, and β-thalassemia. In particularly, the anti-cancer potential of curcumin has been successfully exhibited in various types of cancers namely oral, lung, breast, prostate, pancreatic, colorectal, multiple myeloma and head and neck squamous cell carcinoma. With safety and efficacy proven in more than 65 clinical trials and an additional 35 on-going trials [193], the use of curcumin as a supplement has already been approved in many countries such as the USA, South Africa, India, Nepal, Pakistan, Japan, Korea, China, Thailand, and Turkey. In an attempt to increase its bioavailability, several curcumin formulations have been developed such as powder, tablets, capsules, liposomal encapsulation, emulsions, and nanoparticles [289,290,291]. Apart from being effective by itself, curcumin also functions excellently in combination with various other compounds, such as docetaxel, acetylcysteine, gemcitabine, mesalamine, sulfasalazine, quercetin, pantoprazole, prednisone, bioperine, piperine, lactoferrin, and soy isoflavones [193]. Interestingly, the ramifications of curcumin at the molecular level have also been observed in human trials on the targets described in this review, including NF-κB, STAT3, COX-2, phosphoryl kinase, prostaglandin E2, prostate-specific antigen, transforming growth factor-β, adhesion molecules, pro-inflammatory cytokines, apoptotic proteins, ET-1, 5-LOX, HO-1, C-reactive protein, creatinine, AST, ALT, and triglycerides [193] Numerous pilot clinical studies as well as phase I and II clinical trials of curcumin have been conducted to investigate its anti-cancer potential. These have been summarized in Table 1 and discussed in brief below:

#### **(a) Cancer Lesions** 

The first phase I clinical trial to test the toxicity, pharmacokinetics, and biological effective dose of curcumin was carried out by Cheng *et al*., in 2001 [292]. Twenty-five patients who had either one of the following pre-malignant high cancer risk conditions were included: urinary bladder cancer, arsenic Bowen’s disease of skin, uterine cervical intraepithelial neoplasm (CIN), oral leukoplakia, and intestinal metaplasia of the stomach. An escalating oral dose of 500, 1000, 2000, 4000, 8000, and 12,000 mg was tried and treatment-related toxicity was not observed up to 8000 mg/day. Higher doses were found to be unacceptable to the patients, primarily due to bulky volume of the drug. Peak serum curcumin concentration was reached 1–2 h after intake, while it was undetectable in urine. Histologic improvement of pre-cancerous lesions was observed in one of the two resected bladder cancer patients, two out of six Bowen’s disease patients, one out of four CIN patients, two out of seven oral leukoplakia patients, and one out of six patients with gastrointestinal metaplasia. Unfortunately, frank malignancies developed in one CIN patient and in one oral leukoplakia patient. Thus, in this study, apart from non-toxicity, the chemopreventive potential of curcumin against cancerous lesions was also demonstrated. In another randomized, double blind, placebo-controlled study, the efficacy of curcuminoids was evaluated in 100 patients with oral lichen planus. 2000 mg of curcuminoids or placebo was given every day for 7 weeks and 60 mg/day prednisone was given in the first week [293]. However, the trial was ended prematurely because the interim analysis predicted less than a 2% chance of better outcome in the curcuminoid group compared to placebo. The differences in the results of these two studies could be possibly attributed to the difference in the preparation of the curcumin dose. In a more recent study by Rai *et al.*, 1000 mg curcumin tablet was given daily for 7 days to patients with oral leukoplakia, oral submucous fibrosis, or lichen planus, and also to healthy subjects (25 participants in each group) [294]. Serum and salivary vitamin C and E levels improved and MDA while 8-hydroxydeoxyguanosine (8-OHdG) levels decreased. In addition, these changes were associated with a reported decrease in pain. In a randomized, double blind, placebo controlled trial where 20 subjects were given 6000 mg/day of curcumin in three doses for 14 days, subjects showed improvement in clinical symptoms with a decrease in erythema, ulceration, and Modified Oral Mucositis Index (MOMI) [295].

**Table 1:** Summary of clinical trials with curcumin in various cancers.

Serial	Study	Number of Patients	Health Condition	Dose	Outcome	Reference
1	Double blind crossover	62	Cancer lesions	Topical ointment	Symptomatic relief to patients	[59]
2	Pilot study	16	Lung cancer	1500 mg/day; 30 days (turmeric)	Urinary excretion of mutagens was decreased in smokers	[296]
3	Pilot study	58	Cancer lesions	3600 mg/day; 3 months (turmeric)	Micronuclei number in mucosal cells and circulating lymphocytes decreased	[297]
4	Prospective Phase I trial	25	Cancer lesions	8000 mg/day; 3 months	Histologic improvement of precancerous lesions	[292]
5	Prospective Phase I trial	15	Colorectal cancer	36–180 mg; 4 months	Decrease in glutathione S-transferase activity	[298]
6	Phase I trial	12	Hepatic metastases from colorectal cancer	450–3600 mg/day; 1 week	Low bioavailability of oral dose such that the dose of curcumin required to exert its pharmacological activity at hepatic level is not feasible in humans	[299]
7	Phase I trial	15	Colorectal cancer	450–3600 mg/day; 4 months oral dose	PGE_2_ production reduced	[300]
8	Phase I trial	12	Colorectal cancer	450–3600 mg/day; 7 days	M_1_G levels decreased	[301]
9	Pilot study	5	Colorectal cancer (Familial adenomatous polyposis)	1440 mg/day; 6 months, combined with quercetin	The number and size of polyps reduced without any significant toxicity	[302]
10	Prospective Phase I trial	24	Healthy volunteers	500–1200 mg; single oral dose	Overall well tolerated but 30% subjects had minor adverse events	[303]
11	Randomized, placebo controlled, double blind	100	Cancer lesions in oral lichen planus	2000 mg/day; 7 weeks, combined with prednisone	Not efficacious but well tolerated	[293]
12	Phase I/II trial	29	Multiple myeloma	2000–12,000 mg/day; 12 weeks combined with Bioperine	Well tolerated, improved bioavailability and decrease in NF-κB, COX2 and STAT3	[304]
13	Phase II trial	25	Advanced pancreatic cancer	8000 mg/day; 2 months	Well tolerated but absorption was limited and was effective only in some patients	[305]
14	Single blind, cross over	26	Multiple myeloma	4000 gm/day; 6 months	Urinary *N*-telopeptide of type I collagen and paraprotein levels reduced	[306]
15	Phase I, open-label, dose escalation trial	14	Advanced and metastatic breast cancer	6000 mg/day; 7 days, every 3 weeks, combined with docetaxel	Well tolerated and efficacious	[307]
16	Open-label, phase II trial	17	Advanced pancreatic cancer	8000 mg/day; 4 weeks; combined with gemcitabine	Modest efficacy, therapy not a feasible	[308]
17	Randomized, double blind, controlled	85	Prostate cancer	100 mg/day; 6 months, combined with soy isoflavones	Serum PSA content decreased	[309]
18	Pilot study	75	Pre-cancerous lesions	1000 mg/day; 7 days	MDA and 8-OHdG levels increased in saliva and serum while Vitamin C and E levels reduced	[294]
19	Phase IIa trial	44	Colorectal cancer	2000–4000 mg/day; 1 month	Aberrant Crypt Foci formation reduced only in smokers	[310]
20	Pilot study	126	Colorectal cancer	1080 mg/day; 10–30 days	Increased p53 expression, decrease in serum TNF-α and improved body weight	[233]
21	Phase I/II	21	Gemcitabine-resistant pancreatic cancer	8000 mg/day	Well tolerated	[311]
22	Pilot study	39	Head and neck cancer	2 curcumin tablets	IKKβ kinase activity decreased which correlated with IL-8 decrease in saliva	[312]
23	Randomised, double blind, placebo controlled	20	Cancer lesions in oral lichen planus	6000 mg/day; 14 days	Clinical symptoms reduced with no adverse effects	[295,313]
24	Randomised, double blind, placebo controlled, cross over followed by open label study	36	Multiple myeloma	4000 mg/ day; 3 months followed by 8000 mg/day; 3 months	Slowed down disease progression	[314]
25	Randomized, open label	50	Chronic myeloid leukaemia	15,000 mg/day; 6 weeks in combination with imatinib	Enhanced decrease in nitric oxide levels	[315]

#### **(b) Colorectal Cancer** 

Sharma *et al.*, conducted two phase I trials in colorectal cancer patients who were refractory to conventional chemotherapeutics [298,300]. In the first study in 2001, curcuma extract, equivalent to 36–180 mg of curcumin, was given orally to 15 patients, daily for 4 months [298]. Dose-limiting toxicity was not observed and neither curcumin nor its metabolites were detected in blood or urine but were found in feces. The treatment maintained radiologically stable disease for 2–4 months in five patients. The study indicated safety but low oral bioavailability of curcumin. In a subsequent study in 2004, an escalating dose of curcuminoids comprising 450, 900, 1800, or 3600 mg per day for 4 months was tested in 15 patients [300]. Curcumin and its metabolites were detected in plasma and urine. Overall, the drug was well-tolerated but three patients suffered from minor gastrointestinal adverse events. A minor increase of alkaline phosphate and lactate dehydrogenase was observed in the serum of four and three patients, respectively. Similar minor adverse effects in a few subjects were observed by Lao *et al*., in a prospective phase I trial conducted in 2006 [303]. In this study, 24 healthy volunteers were given escalating doses of 500, 1000, 2000, 4000, 8000, 10,000 and 12,000 mg in a single dose and safety was assessed 72 h later. 30% of the subjects experienced diarrhea, headache, rash, and yellowish stools irrespective of the dose.

Garcea *et al.*, investigated whether pharmacologically active levels of curcumin could be achieved in the colon and rectum of colorectal cancer patients [299]. After oral administration of 450, 1800, or 3600 mg of curcumin every day for 7 days, traces of curcumin and its metabolites were found in normal mucosa, malignant colorectal tissues, and intestinal tissues. The observed decrease in malondialdehyde-DNA M1G-adduct and COX-2 in colorectal tissues suggested efficacy of the dosage. Even in combination therapy, wherein 480 mg of curcumin was given with 20 mg of quercetin thrice a day, the number and size of polyps in patients suffering from familial adenomatous polyposis was found to be reduced after 6 months of treatment [302]. In a non-randomized, open-label, phase IIa trial, 44 smokers with eight or more aberrant crypt foci (ACF), which are a precursor of colorectal polyps, were treated with 2000 or 4000 mg of curcumin daily for 30 days [310]. Only the higher dose significantly reduced ACF formation, which was associated with a five-fold increase in post-treatment plasma curcumin/conjugate levels. However, the underlying mechanism is yet unclear. In another study, 360 mg of curcumin was administered thrice a day for 10–30 days in colorectal cancer patients after diagnosis and before surgery [233]. Curcumin administration resulted in an increase in body weight, number of apoptotic cells, and p53 expression, while it decreased TNF-α level in serum. The study concluded that curcumin treatment can improve the general health of colorectal cancer patients, probably, but not necessarily, due to the increase in p53 expression.

#### **(c) Multiple Myeloma** 

The chemopreventive potential of curcumin in monoclonal gammopathy of undetermined significance (MGUS), a high-risk condition for progression to multiple myeloma, was tested by Golombick *et al*., in a cross-over study design [306]. Out of 27 patients, 17 received 4000 mg of curcumin for 3 months before cross-over to placebo. The rest received placebo first, followed by curcumin. The levels of paraprotein and urinary N-telopeptide from type I collagen decreased in some patients. The same group reported beneficial effects of curcumin in MGUS in another randomized, double blind, placebo-controlled cross-over study [314]. A phase I/II trial by Vadhan-Raj *et al*., in 29 patients with asymptomatic, relapsed, or plateau phase multiple myeloma showed a decrease in expression of NF-κB, COX-2, and STAT3 in peripheral blood mononuclear cells and stable maintenance of disease [304]. The patients were given either oral dose of curcumin alone (2000, 4000, 6000, 8000, or 12,000 mg/day) or in combination with 10 mg of bioperine. 12 patients continued the treatment for 12 weeks, followed by combination treatment for five patients (one at a dose of 4000, two at 6000 mg, and two at 8000 mg) for 1 year. Further, well-controlled clinical trials with larger sample sizes are required to substantiate the efficacy of curcumin against multiple myeloma.

#### **(d) Pancreatic Cancer** 

Dhillon *et al.*, conducted a phase II clinical trial in 25 advanced pancreatic cancer patients [305]. An oral dose of 8000 mg of curcumin was given every day until disease progression, with restaging performed every 2 months. Low, steady-state levels of curcumin and its conjugates were detected in the peripheral circulation for the first 4 weeks with a peak at 22–42 ng/mL. In two patients, a biological effect was seen and in one the disease was maintained stably for 18 months. Remarkably, a significant but brief tumor regression was seen in one patient with a concomitant increase in serum cytokine levels. NF-κB, COX-2, and STAT3 suppression in peripheral mononuclear cells was also observed in the patients. Although oral curcumin was found to be safe and well-tolerated, poor bioavailability still remains an issue, which partly explains the biologic activity observed only in some patients. In another open-label, phase II trial, a combination of curcumin and gemcitabine was tested in advanced pancreatic cancer patients [308]. Seventeen patients were administered 8000 mg of curcumin orally every day for 4 weeks while 1000 mg/m^3^ of gemcitabine were given intravenously three times a week. In five patients, curcumin or the whole treatment was discontinued due to toxicity and one patient died suddenly. In the remaining 11 patients, a partial response was seen; four had stable disease while in six the tumor progressed. Tumor progression time was 1–12 months, with overall survival time of 1–24 months, indicating a modest efficacy of the combination therapy. Also, the authors concluded that 8000 mg/day of curcumin with gemcitabine was above the maximum tolerated dose. In a recent phase I/II trial by Kanai *et al*., a similar combination of gemcitabine and curcumin was used to treat gemcitabine-resistant pancreatic cancer in 21 patients [311]. In contrast, the combination of gemcitabine and curcumin 8000 mg/day was found to be safe and well tolerated in this study. Studies in a larger cohort are required to validate the results.

#### **(e) Breast Cancer** 

An open-label, phase I clinical trial was conducted by Bayet-Robert *et al*., to evaluate the feasibility and tolerability of curcumin in combination with docetaxel [307]. Fourteen patients with advanced or metastatic breast cancer were given 100 mg/m^3^ of docetaxel every 3 weeks on day 1 for six cycles, while an oral escalating dose of curcumin was given for seven consecutive days, starting from 500 mg/day until a dose-limiting toxicity was observed. The authors reported that the maximum tolerable dose was 8000 mg/day but they advocated 6000 mg/day for 7 consecutive days every 3 weeks in combination with the standard docetaxel dose for treatment of breast cancer.

#### **(f) Prostate Cancer** 

Ide *et al.*, conducted a randomized, double blind study, wherein the efficacy of a combination therapy of soy isoflavones and curcumin was evaluated [309]. Eighty five participants who underwent prostate biopsies because of increased PSA (prostate-specific antigen) levels but had negative prostate cancer findings were enrolled. Either a supplement containing 40 mg of isoflavones and 100 mg of curcumin or placebo was administered daily for six months. Serum PSA levels decreased in the combination therapy group. The authors suggested a possible synergistic role of curcumin with soy isoflavones in the suppression of PSA production. The anti-cancer activity of curcumin via inhibition of IKKβ kinase activity was examined in patients with head and neck squamous cell carcinoma (HNSCC) [312]. 13 patients with dental carries, 21 with HNSCC, and five healthy volunteers (total 39) were given two tablets, which they chewed for 5 min. IKKβ kinase activity was suppressed in salivary cells while IL-8 levels decreased in patients with dental carries, but the decrease in IL-8 levels was not significant in HNSCC patients. The authors suggested the use of IKKβ kinase as a biomarker for assessing the effect of curcumin in HNSCC.

#### **(g) Chronic Myeloid Leukemia** 

Recently, in a randomized open label study, the efficacy and safety of curcumin was evaluated against chronic myeloid leukemia [315]. 25 participants were given 800 mg of imatinib daily for 6 weeks while the rest were given a combination of 800 mg of imatinib with 5000 mg of curcumin thrice a day. Nitric oxide levels were found to decrease significantly in the combination therapy group, as compared to placebo and imatinib only group. The authors advocated the use of curcumin as an adjuvant to imatinib for the treatment of chronic myeloid leukemia.

## 4. Conclusions

A plethora of *in vitro* and *in vivo* research together with clinical trials conducted over the past few decades substantiate the potential of curcumin as an anti-cancer agent. At the molecular level, curcumin targets numerous pathways, highlighting its ability to inhibit carcinogenesis at multiple levels and thus, potentially circumventing the development of resistance. However, there is a paucity of data to explain the underlying mechanism of its activity. Clinical trials with curcumin indicate safety, tolerability, non-toxicity (even up to doses of 8000 mg/day), and efficacy. These studies provide a solid foundation for more well-controlled studies in larger cohorts as well as open avenues for future drug development. However, curcumin activity is limited by its poor bioavailability and some possible adverse effects. The development of formulations of curcumin in the form of nanoparticles, liposomes, micelles or phospholipid complexes to enhance its bioavailability and efficacy are still in its early stages. Nonetheless, curcumin has established itself as a safe and promising molecule for the prevention and therapy of not only cancer but also other inflammation-driven diseases.

## Abbreviation

AP-1: activator protein 1
AAPK: Abscisic-acid-activated protein kinase
AHR: aryl hydrocarbon receptor
AR: androgen receptor
AK: adenylate kinase
ATPase: adenosine triphosphatases
Bad: Bcl2 associated death promoter protein
Bid: BH3 interacting-domain death agonist
Bcl2: B-cell lymphoma 2
Bcl-xL: B-cell lymphoma extra-large
Bax: Bcl-2-associated X protein
CTGF: connective tissue growth factor
CXCR: chemokine receptor
CXCL: chemokine ligand
Ca2+PK: calcium/calmodulin dependent protein kinase
CREB-BP: CREB-binding protein
CCND1: Cyclin D1
COX-2: cyclooxygenase-2
c-myc: Myelocytomatosis cellular oncogene
DR: death receptor
DEF-40: Differentially Expressed In FDCP
EGFR: epidermal growth factor receptor
EGFR-K: Epidermal growth factor receptor-kinase
ERK: extracellular-signal-regulated kinases
ECM: extracellular matrix
ER-α: estrogen receptor-alpha
EPCR: Endothelial protein C receptor
ELAM-1: endothelial-leukocyte adhesion molecule-1
EGF: epidermal growth factor
ERE: estrogen response element
EGR-1: early growth response
FAK: Focal Adhesion Kinase
vFLIP: viral FADD-like interleukin-1β-converting enzyme (FLICE)/caspase-8-inhibitory protein
FGF: fibroblast growth factor
Fas R: Fas receptor/CD95
FPT: Farnesyl protein transferase
GST: glutathione-S-transferase Telomerase
GCL: Glutamate Cysteine Ligase
HGF: hepatocyte growth factor
HIF-1α: hypoxia-inducible factor 1α
H2R: histamine H2 receptor
HER-2: human epidermal growth factor receptor 2
Hsp-70: Heat shock protein-70
ICAM-1: intracellular adhesion molecule 1
IL: interleukin
IGF-1: insulin-like growth factor
IκB: inhibitory κB
IKK: IκB kinase
IAP-1: inhibitor of apoptosis
IR: insulin receptor
ITR: Inositol triphosphate receptor
iNOS: inducible nitric oxide synthase
JAK: Janus kinase
LDLR: Low-Density Lipoprotein receptor
5-LOX: 5-lipoxygenase
MMP: matrix metalloproteinase
MCP: monocyte chemoattractant protein
MIP: macrophage inflammatory protein
MDRP: multidrug resistant protein
MAPK: mitogen-activated protein kinase
NF-κB: nuclear factor κB
Nrf-2: Nuclear factor (erythroid-derived 2)-like 2
NGF: nerve growth factor
NSCLC: non-small cell lung cancer
NQO-1: NAD(P)H:quinone acceptor oxidoreductase 1
ODC: ornithine decarboxylase
PTK: protein tyrosine kinase
PKA: protein kinase A
PKB: protein kinase B/AKT
PhK: Phosphorylase kinase
PAK: p21 activated kinases
Pp60C-TK: pp60c-src tyrosine kinase
PDGF: platelet derived growth factor
p53: tumor suppressor gene
PPAR-γ: peroxisome proliferator associated receptor gamma
cPLA2: Secreted phospholipases A2
PI3K: Phosphoinositide-3-kinase
RCC: renal cell carcinoma
ROS: reactive oxygen species
Src-2: src-non receptor tyrosine kinase
STAT3: signal transducer and activator of transcription 3
SCC: squamous cell carcinoma
TNF: tumor necrosis factor
TF: tissue factor
TGF-β1: transforming growth factor-beta
TMMP-3: truncated matrix metalloprotease-3
TAM: tumor-associated macrophage
uPA: urokinase plasminogen activator
VEGF: vascular endothelial growth factor
VCAM-1: vascular cell adhesion molecule 1
WT-1: Wilms tumor-1
XIAP: X-linked inhibitor of apoptosis
